# The Efficacy of WeChat-Based Parenting Training on the Psychological Well-being of Mothers With Children With Autism During the COVID-19 Pandemic: Quasi-Experimental Study

**DOI:** 10.2196/23917

**Published:** 2021-02-10

**Authors:** Guihua Liu, Shuo Wang, Jinhua Liao, Ping Ou, Longsheng Huang, Namei Xie, Yingshuang He, Jinling Lin, Hong-Gu He, Rongfang Hu

**Affiliations:** 1 The School of Nursing Fujian Medical University Fuzhou China; 2 Department of Child Healthcare Centre Fujian Provincial Maternity and Child Health Hospital, Affiliated Hospital of Fujian Medical University Fuzhou China; 3 Alice Lee Centre for Nursing Studies Yong Loo Lin School of Medicine National University of Singapore Singapore Singapore; 4 National University Health System Singapore Singapore

**Keywords:** coronavirus disease 2019, autism spectrum disorder, parenting training, psychological well-being, social media, WeChat, COVID-19, autism, parenting, mental health, well-being, anxiety, depression, stress

## Abstract

**Background:**

During the COVID-19 pandemic, special education schools for children in most areas of China were closed between the end of January and the beginning of June in 2020. The sudden interruption in schooling and the pandemic itself caused parents to be anxious and even to panic. Mobile-based parenting skills education has been demonstrated to be an effective method for improving the psychological well-being of mothers with children with autism. However, whether it can improve the psychological states of mothers in the context of the COVID-19 pandemic is a subject that should be urgently investigated.

**Objective:**

The aim of this study is to evaluate the efficacy of WeChat-based parenting training on anxiety, depression, parenting stress, and hope in mothers with children with autism, as well as the feasibility of the program during the COVID-19 pandemic.

**Methods:**

This was a quasi-experimental trial. A total of 125 mothers with preschool children with autism were recruited in January 2020. The participants were assigned to the control group (n=60), in which they received routine care, or the intervention group (n=65), in which they received the 12-week WeChat-based parenting training plus routine care, according to their preferences. Anxiety, depression, parenting stress, hope, satisfaction, and adherence to the intervention were measured at three timepoints: baseline (T0), postintervention (T1), and a 20-week follow-up (T2).

**Results:**

In total, 109 mothers completed the T1 assessment and 104 mothers completed the T2 assessment. The results of the linear mixed model analysis showed statistically significant group × time interaction effects for the intervention on anxiety (*F*=14.219, *P*<.001), depression (*F*=26.563, *P*<.001), parenting stress (*F*=68.572, *P*<.001), and hope (*F*=197.608, *P*<.001). Of all mothers in the intervention group, 90.4% (48.8/54) reported that they were extremely satisfied with the WeChat-based parenting training. In total, 40.0% (26/65) logged their progress in home training each week and 61.5% (40/65) logged their progress more than 80% of the time for all 20 weeks.

**Conclusions:**

The WeChat-based parenting training is acceptable and appears to be an effective approach for reducing anxiety, depression, and parenting stress, as well as increasing hope in mothers with children with autism during the global COVID-19 pandemic. Future studies with rigorous designs and longer follow-up periods are needed to further detect the effectiveness of the WeChat-based parenting training.

**Trial Registration:**

Chinese Clinical Trial Registry ChiCTR2000031772; http://www.chictr.org.cn/showproj.aspx?proj=52165

## Introduction

COVID-19 emerged in Wuhan in late 2019 and then spread throughout China. Now, it has swept through more than 200 countries and has been identified as a public health emergency of international concern and characterized as a pandemic by the World Health Organization (WHO) [1]. COVID-19 is caused by SARS-CoV-2, which is genetically similar to severe acute respiratory syndrome coronavirus (SARS-CoV). In addition, it seems to be less pathogenic but more transmissible than SARS-CoV and Middle East respiratory syndrome coronavirus (MERS-CoV) [2-4]. The Chinese government initiated a first-level public health response to prevent the spreading of the outbreak. For example, the city of Wuhan was locked down on January 23, 2020, and the government implemented compulsory measures that restricted gatherings [5]. Nationwide school closures were implemented by the Ministry of Education, and 47 million preschool children were confined to their homes until the outbreak was mostly under control [6]. Most of the special education schools in China resumed classes at the beginning of June. This closure period lasted for about four months or even longer. As such, these preschool children were not able to engage in various forms of learning activities. The sudden interruption in learning and the impacts of the pandemic itself caused anxiety and even panic among parents.

Autism spectrum disorder (ASD) is a heterogeneous neurodevelopmental disorder typically characterized by restricted interests, repetitive behaviors, and deficits in social reciprocity and communication [7]. These impairments not only affect children’s physical development but also their mental development, especially social-emotional development, which may result in their parents experiencing psychological stress and economic burden [8]. In recent years, the prevalence rate of children with ASD has been as high as 10.18 per 10,000 and has been increasing in China [9]. Children with ASD require continuous long-term training to improve their cognitive development and behaviors. Due to the deficits in social reciprocity and communication, the special training teachers, regular training activities, and training place for each child are relatively fixed. However, the sudden COVID-19 outbreak interrupted familiar and routine training activities for preschool children with ASD, and restrictions of the children’s physical environments may exacerbate their behavioral problems [10]. Coupled with the impacts of the pandemic, parents, especially mothers, who may have already been under great psychological pressure may become more helpless and hopeless. Additional support should be provided to individuals with ASD who are identified to be in a group with higher risk of complications from COVID-19. Furthermore, support for the mental well-being of families is essential during the outbreak to avoid increases in parental stress [11].

Parenting training, a promising approach for teaching specific techniques and strategies, is recommended for parents with children with autism; such techniques can include parent-mediated social communication therapy and parent-child joint engagement [12,13]. However, access to face-to-face support for parents is often restricted in China due to factors such as parents’ time constraints, distance, and uncontrollable weather conditions. According to the WHO, telehealth interventions can deliver health care using telecommunications and virtual technologies [14]. Given the advantages of telehealth interventions (eg, low cost, easy dissemination, and high accessibility), they can be used in parental training [15]. In addition, telehealth interventions can ensure the consistency of teachers and places, and the continuity of the intervention, which may be more suitable for children with ASD—and their parents—who lack access to face-to-face training. Recent studies have shown that telehealth interventions can improve the behaviors of children with ASD [16-19]. Telehealth interventions based on computer and internet technologies have also served as effective methods for improving the psychological well-being of mothers with children with autism [17,20]. However, whether mobile phone–based parenting training can improve the psychological states of mothers in the context of a pandemic is an urgent subject to be investigated. Therefore, this study aimed to evaluate the efficacy of WeChat-based parenting training on the psychological well-being of mothers with children with autism during the COVID-19 pandemic. We had the following hypotheses:

1. The WeChat-based parenting training program is feasible and acceptable for mothers with children with autism.
2. When compared to those in the control group, mothers in the intervention group will report lower levels of anxiety, depression, and parenting stress, and higher levels of hope during the COVID-19 pandemic.

## Methods

### Study Design

An assessor-blind quasi-experimental trial with a nonequivalent control group and nonrandom distribution was conducted. The study protocol was formulated to standardize the study.

### Ethical Considerations

Ethical approval was obtained from the Research Ethics Committee of Fujian Medical University and the study hospital (Fujian Provincial Maternity and Child Health Hospital; 2017-105). All participants were informed that participation was voluntary and that they could refuse to participate in or withdraw from the study at any time without negative consequences to any other treatments. Written informed consent was obtained from each participant in the form of photographs. The data were kept anonymous and confidential and were only used for this study.

### Setting and Sample

We enrolled the participants from several campuses of a special education school in Fuzhou, China. Mothers with preschool children with autism were recruited in January 2020. The original purpose of the study was to verify the effectiveness of the WeChat-based parenting training. However, as the COVID-19 pandemic occurred before recruitment was completed, the purpose of this study was adjusted to evaluate the effectiveness and feasibility of the WeChat-based parenting training in the context of a pandemic. Considering the pandemic situation, after a consultation with the hospital ethics committee, the participants were allowed to choose their group according to their preferences. Although a prospective quasi-experimental study with nonrandom distribution is less rigorous than a randomized controlled trial design, a parallel control group was used to make the study methodology as rigorous as possible. Assessor blinding was performed through the Questionnaire Star platform.

The inclusion criteria were the following: (1) mothers who were the primary caregivers of preschool children aged 3-7 years old who were diagnosed with ASD according to the Diagnostic and Statistical Manual of Mental Disorders, Fifth Edition (DSM-5), (2) mothers who owned a smartphone and had a WeChat account and were willing to enroll in a training class with their WeChat ID, and (3) mothers who were able to read and understand Mandarin Chinese.

The exclusion criteria were the following: (1) mothers whose children were diagnosed with Rett syndrome, childhood disintegrative disorder, or other terminal illnesses, (2) mothers whose children (and/or mothers themselves) had received psychosocial treatments (such as mindfulness-based training, journal writing, and parent-mediated social communication therapy) in the past 8 months, and (3) mothers whose children (or mothers themselves) were diagnosed with COVID-19 or terminal illnesses during the intervention.

### Sample Size

G*Power (Version 3.1.9.6; Franz Faul, Universität Kiel) was used to calculate the sample size. Assuming a power of 0.80, an α of 0.05, and an effect size of 0.56 for parenting stress based on a preliminary test, the corrected sample size was 60 per group with a 15% dropout rate. We recruited 125 participants.

### Interventions

#### Control Group

The mothers in the control group received routine care, which included the following:

1. An electronic manual entitled “108 Strategies to Overcome the Pandemic at Home” [21], organized by the Fujian Provincial Health Commission and the Fujian Provincial Press and Publication Bureau and compiled by members of the research team, was uniformly distributed to guide families through the pandemic at home. The contents of the manual included six modules: home protection, outside protection, diet, coping strategies for common problems, medical guidance, and parent-child games and sports. A comic cartoon was inserted into the manual to facilitate parent-child reading and understanding. The manual was also interspersed with animations and other videos in the form of two-dimensional codes for intuitive interpretations.
2. A home training plan for children was distributed to the parents and training progress was checked in the form of homework once per week for 12 weeks.

#### Intervention Group

The mothers in the intervention group received routine care plus the WeChat-based parenting training. The WeChat-based parenting training included the following:

1. The Joint Attention, Symbolic Play, Engagement, and Regulation (JASPER) online course delivered via WeChat [13]. The JASPER course focused on targeted social communication strategies in the format of parent-child coaching sessions that went on for 45-60 minutes per session, with two sessions each week for 12 weeks. Specific strategies for high-quality responses to children’s communication and behaviors were provided by one special training teacher with more than five years of special training work experience. Another teacher was responsible for demonstrating any scenario simulations.
2. An online question-and-answer session. A question-and-answer session (30-40 minutes) was conducted each week for 12 weeks.
3. An online parental psychological intervention course based on pandemic situations. The course was conducted by team researchers with second-level psychological counseling qualifications. The contents included home protection strategies, emotional management, parental stress coping strategies, and psychological counseling strategies to cope with the pandemic situation (eg, mindfulness breathing training, muscle relaxation training, and the traditional Chinese Qigong exercise “Ba Duan Jin”) and lasted 45-60 minutes per session, with one session every two weeks and 6 sessions in total. For all online courses, live links were generated by the class assistant software Little Goose (Shenzhen Xiao’e Network Technology Co) and then sent to the WeChat group.

### Outcome Measurements

#### Primary Outcomes

##### Anxiety

The Self-rating Anxiety Scale (SAS) [22] was used to assess the mothers’ anxiety levels. The scale has 20 items that are measured by a 4-point Likert-type scale, ranging from 1 (none or a little of the time) to 4 (most or all of the time), with a total score of 20-80. A higher score indicates a higher anxiety level. The total score is then multiplied by 1.25 to get a standard score. A standard score of <50 indicates no anxiety, 50-59 indicates mild anxiety, 60-69 indicates moderate anxiety, and ≥70 indicates severe anxiety. Good validity and reliability were demonstrated when the SAS was used in China [23]. In this study, the total Cronbach α value was 0.882 and the rest-retest reliability was 0.872.

##### Depression

The Self-rating Depression Scale (SDS) was used to measure the mothers’ depression levels [24]. The scale has 20 items that are measured on a 4-point Likert-type scale, with a total score ranging from 20 to 80. The total score is multiplied by 1.25 to obtain a standard score. A standard score of <53 indicates no depression, 53-62 indicates mild depression, 63-72 indicates moderate depression, and ≥73 indicates severe depression. The Chinese version of the SDS was validated in the Chinese population with good reliability [25]. In this study, the total Cronbach α value was 0.838 and the rest-retest reliability was 0.818.

##### Parenting Stress

The Parenting Stress Index-Short Form (PSI-SF) [26] was used to measure parenting stress. The scale has 20 items that are measured by a 5-point Likert-type scale, ranging from 1 (strongly disagree) to 5 (strongly agree), with a total score ranging from 36 to 180. The PSI-SF comprises three dimensions: parenting distress, parent-child dysfunctional interaction, and difficult child. A total score on the PSI-SF above 90 (≥90th percentile) indicates that further professional intervention is required [26]. The original version has a good internal consistency, with Cronbach α values ranging from 0.80 to 0.91, as well as good rest-retest reliability (*r*=0.68-0.85), and the Chinese version was also validated with good psychometric properties [26-28]. In this study, the total Cronbach α value was 0.899 and the test-retest reliability was 0.801.

#### Secondary Outcomes

##### Hope

Hope levels were assessed by the Herth Hope Index (HHI), a 12-item scale containing three dimensions: temporality and future, positive readiness and expectancy, and interconnectedness [29]. The scale adopts a 4-point Likert-type scale, ranging from 1 (completely disagree) to 4 (completely agree), with a maximum total score of 48. A total score of 12-23 indicates a low level of hope, 24-35 indicates a medium level of hope, and 36-48 indicates a high level of hope. The Chinese version of the HHI has appropriate internal consistency, content validity, and convergent and discriminant validity [30]. In this study, the total Cronbach α value was 0.762 and the rest-retest reliability was 0.837.

##### Feasibility

Feasibility measures included acceptability and demand. Acceptability was measured with a postintervention satisfaction survey in the intervention group. A total of five self-reported statements for the satisfaction assessment in the questionnaire are answered with ratings of “strongly disagree,” “slightly disagree,” “neutral,” “slightly agree,” and “strongly agree.” Demand was measured using adherence to the WeChat-based parenting training intervention. Adherence to the intervention was recorded in weekly reports from the WeChat progress log for home training using the WeChat mini-program (a “sub-app” based on the WeChat platform that can be used without installation) for each participant.

### Data Collection

Data were collected through the survey invitation links generated by the Questionnaire Star platform (Wenjuanxing [31]) at baseline (T0), on the second or third day after the intervention (T1, Week 12), and two months after the intervention (T2, Week 20). The assistants (who were blinded to the assignments) collected the participants’ demographic characteristics and measured the outcomes from the platform to reduce bias.

### Statistical Analysis

IBM SPSS (Version 25.0; IBM Corp) and GraphPad Prism (Version 8.0.2; GraphPad Software Inc) were used to analyze the data and prepare the figures. An intention-to-treat analysis (ITT) and a linear interpolation were performed. Descriptive statistics, such as mean, standard deviation, frequency, and percentage, were used to report the demographic and outcome variables. Independent two-sample *t* tests, chi-square tests, and Mann-Whitney *U* tests were used to compare the baseline characteristics between the two groups. A linear mixed model was used to examine group effects, time effects, and group × time interaction effects on anxiety, depression, parenting stress, and hope levels over time. Cohen *d* effect size analysis was performed to compare the magnitude of the effects at each timepoint, and 0.2, 0.5, and 0.8 were considered as small, medium, and large effects, respectively [32].

## Results

### Participant Enrollment

**Figure 1:**
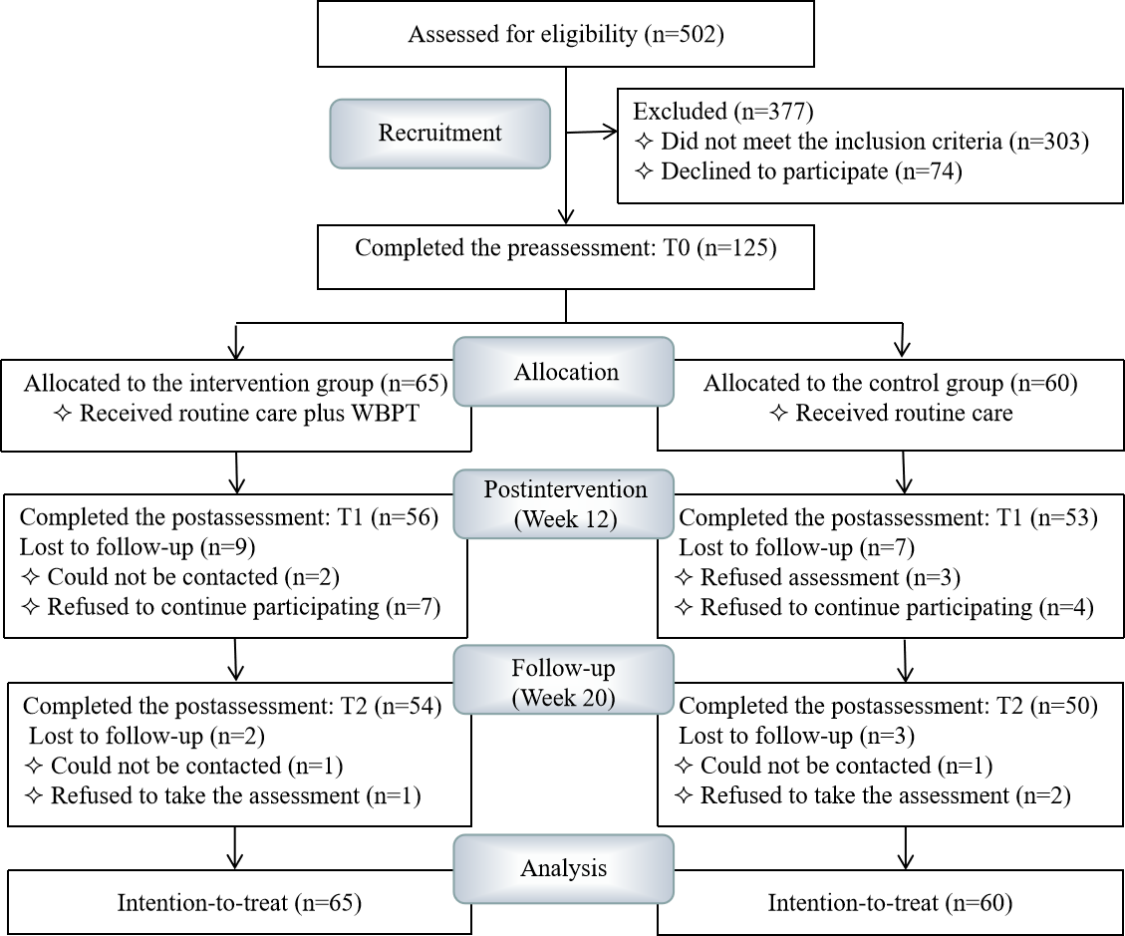
CONSORT flow chart of the study. CONSORT: Consolidated Standards of Reporting Trials; WBPT: WeChat-based parenting training.

Among the 502 mothers who were approached, 303 of them did not meet the inclusion criteria (230 mothers whose child was diagnosed with mental retardation, 55 with language retardation, 5 with attention deficit and hyperactivity disorder, 5 with developmental coordination disorder, 3 with Rett syndrome, 3 with Down syndrome, and 2 with medically active diseases) and 74 mothers declined to participate in the study (67 with schedule conflict and 7 who refused to do the preassessment). Eventually, 125 mothers were recruited and assigned to one of two groups (60 in the control group and 65 in the intervention group) based on their preferences. After a 20-week research period, 7 mothers were uncontactable, and 14 mothers refused to participate in the assessments. In total, 104 mothers completed the follow-up assessments (Figure 1).

### Participants’ Characteristics

There were no significant differences in the participants’ characteristics between the two groups at baseline (all *P*>.05). The participants’ demographic and clinical characteristics are presented in Table 1.

**Table 1:** Participants’ baseline characteristics (N=125).

Variables			Total (n=125), n (%)	Control (n=60), n (%)	WeChat-based parenting training (n=65), n (%)	*χ*^2^*/Z/t* values	*P* value
**Mothers and family**							
	**Age (years), mean (SD)**		32.89 (3.68)	32.57 (3.45)	33.18 (3.89)	0.937	.35^a^
	**Number of children**					–0.879	.38^b^
		1	93 (74.4)	47 (78.4)	46 (70.8)	N/A^c^	N/A
		2	29 (23.2)	11 (18.3)	18 (27.7)	N/A	N/A
		≥3	3 (2.4)	2 (3.3)	1 (1.5)	N/A	N/A
	**Residence**					0.020	.89^d^
		Urban	112 (89.6)	54 (90.0)	58 (89.2)	N/A	N/A
		Rural	13 (10.4)	6 (10.0)	7 (10.8)	N/A	N/A
	**Marital status**						.67^e^
		Married	120 (96.0)	57 (95.0)	63 (96.9)	N/A	N/A
		Divorced/separated	5 (4.0)	3 (5.0)	2 (3.1)	N/A	N/A
	**Occupation**					0.937	.33^d^
		Employed	92 (74.4)	47 (78.3)	46 (70.8)	N/A	N/A
		Unemployed	32 (25.6)	13 (21.7)	19 (29.2)	N/A	N/A
	**Religious belief**						.17^e^
		Yes	116 (92.8)	58 (96.7)	58 (89.2)	N/A	N/A
		No	9 (7.2)	2 (3.3)	7 (10.8)	N/A	N/A
	**Education**					–0.376	.71^b^
		Junior middle school or below	25 (20.0)	12 (20.0)	13 (20.0)	N/A	N/A
		High school/technical secondary school	37 (29.6)	19 (31.7)	18 (27.7)	N/A	N/A
		College	39 (31.2)	18 (30.0)	21 (32.3)	N/A	N/A
		University degree	20 (16.0)	10 (16.7)	10 (15.4)	N/A	N/A
		Graduate degree or above	4 (3.2)	1 (1.6)	3 (4.6)	N/A	N/A
	**Average monthly household income, ¥ (US $)**					–0.120	.90^b^
		<2000 (<310)	10 (8.0)	4 (6.7)	6 (9.2)	N/A	N/A
		2000-5999 (310-929)	51 (40.8)	26 (43.3)	25 (38.5)	N/A	N/A
		6000-10,000 (929-1549)	38 (30.4)	18 (30.0)	20 (30.8)	N/A	N/A
		>10,000 (>1549)	26 (20.8)	12 (20.0)	14 (21.5)	N/A	N/A
	**Family structure**					0.161	>.99^e^
		Nuclear family (parents and minor children living together)	39 (31.2)	19 (31.7)	20 (30.8)	N/A	N/A
		Other immediate family (grandparents and nuclear)	82 (65.6)	39 (65.0)	43 (66.1)	N/A	N/A
		Single-parent family	4 (3.2)	2 (3.3)	2 (3.1)	N/A	N/A
**Children**							
	**Gender**					0.012	.91^d^
		Male	89 (71.2)	43 (71.7)	46 (70.8)	N/A	N/A
		Female	36 (28.8)	17 (28.3)	19 (29.2)	N/A	N/A
	Age (years), mean (SD)		4.52 (1.19)	4.72 (1.14)	4.34 (1.06)	–1.241	.22^a^
	Disease course (years), mean (SD)		1.58 (1.10)	1.70 (1.82)	1.46 (1.82)	0.228	.82^a^
	Childhood Autism Rating Scale score, mean (SD)		32.07 (1.82)	32.03 (1.21)	32.11 (1.16)	–1.770	.08^a^

^a^*T* test.

^b^Mann-Whitney *U* test.

^c^N/A: not applicable.

^d^*χ*^2^ test.

^e^Fisher exact test.

### Primary Outcomes

#### Anxiety

**Figure 2:**
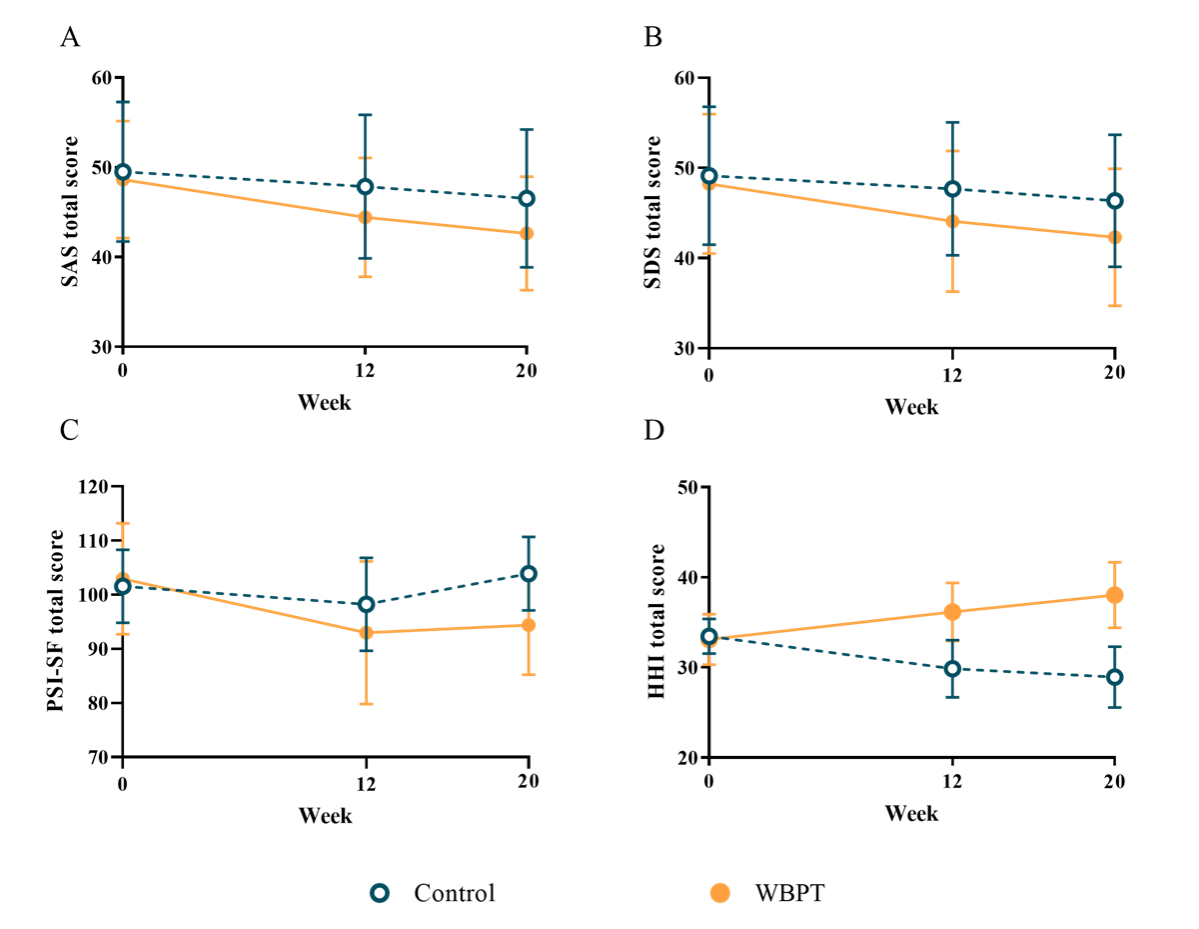
Means and 95% CIs for SAS, SDS, PSI-SF, and HHI scores from baseline to follow-up. HHI: Herth Hope Index; PSI-SF: Parenting Stress Index-Short Form; SAS: Self-rating Anxiety Scale; SDS: Self-rating Depression Scale; WBPT: WeChat-based parenting training.

The results of the linear mixed model analysis indicated a significant group effect (*F*=4.906, *P*=.029), a significant time effect (*F*=93.760, *P*<.001), and a significant group × time interaction effect (*F*=14.219, *P*<.001) on the SAS (Table 2). A significant difference was observed between the two groups at T1 (Cohen *d*=–0.465) and T2 (Cohen *d*=–0.556) for the SAS (Figure 2A).

**Table 2:** Comparisons of scores between groups over time using a linear mixed model (N=125).

Variables and groups			Baseline (T0)	Week 12 (T1)^a^	Week 20 (T2)^a^	Group	Time	Time × group
			Mean (SD)	Mean (SD)	Mean (SD)	*F* value (*P* value)	*F* value (*P* value)	*F* value (*P* value)
**Self-rating Anxiety Scale**								
	Control (n=60)		49.52 (7.78)	47.85 (8.02)	46.53 (7.67)	4.906 (.03)	93.760 (<.001)	14.219 (<.001)
	WBPT^b^ (n=65)		48.65 (6.53)	44.44 (6.63)	42.64 (6.32)	N/A^c^	N/A	N/A
**Self-rating Depression Scale**								
	Control (n=60)		49.13 (7.66)	47.69 (7.38)	46.36 (7.33)	4.457 (.04)	154.830 (<.001)	26.563 (<.001)
	WBPT (n=65)		48.24 (7.72)	44.09 (7.80)	42.32 (7.60)	N/A	N/A	N/A
**Parenting Stress Index-Short Form**								
	**Parenting distress**							
		Control (n=60)	35.49 (3.23)	34.88 (2.95)	35.45 (3.96)	4.083 (.045)	53.994 (<.001)	38.792 (<.001)
		WBPT (n=65)	36.31 (4.29)	33.57 (3.97)	32.09 (3.62)	N/A	N/A	N/A
	**Parent-child dysfunctional interaction**							
		Control (n=60)	34.88 (2.95)	32.31 (3.07)	33.60 (3.78)	6.350 (.01)	34.508 (<.001)	60.743 (<.001)
		WBPT (n=65)	33.57 (3.97)	30.48 (4.46)	28.80 (4.14)	N/A	N/A	N/A
	**Difficult child**							
		Control (n=60)	35.45 (3.96)	34.01 (3.40)	34.82 (4.07)	2.507 (.12)	11.629 (<.001)	.741 (.45)
		WBPT (n=65)	32.09 (3.62)	32.74 (4.63)	33.52 (4.28)	N/A	N/A	N/A
	**Parenting Stress Index-Short Form total score**							
		Control (n=60)	101.56 (6.77)	98.22 (8.59)	103.88 (6.82)	8.176 (.005)	69.315 (<.001)	68.572 (<.001)
		WBPT (n=65)	102.92 (10.27)	92.98 (13.18)	94.41 (9.17)	N/A	N/A	N/A
**Herth Hope Index**								
	**Temporality and future**							
		Control (n=60)	10.75 (1.24)	9.60 (1.32)	9.23 (1.41)	49.276 (<.001)	1.685 (.12)	85.808 (<.001)
		WBPT (n=65)	10.67 (1.27)	11.53 (1.51)	12.23 (1.75)	N/A	N/A	N/A
	**Positive readiness and expectancy**							
		Control (n=60)	11.46 (1.03)	10.01 (1.44)	9.70 (1.35)	74.080 (<.001)	2.228 (.11)	89.652 (<.001)
		WBPT (n=65)	11.28 (1.28)	12.21 (1.35)	12.76 (1.44)	N/A	N/A	N/A
	**Interconnectedness**							
		Control (n=60)	11.25 (1.12)	10.25 (1.84)	10.00 (2.02)	50.344 (<.001)	5.690 (.004)	67.373 (<.001)
		WBPT (n=65)	11.16 (1.33)	12.42 (1.46)	13.05 (1.53)	N/A	N/A	N/A
	**Herth Hope Index total score**							
		Control (n=60)	33.46 (1.92)	29.86 (3.18)	28.93 (3.39)	105.581 (<.001)	4.787 (.009)	197.608 (<.001)
		WBPT (n=65)	33.11 (2.81)	36.16 (3.22)	38.04 (3.62)	N/A	N/A	N/A

^a^Linear interpolation.

^b^WBPT: WeChat-based parenting training.

^c^N/A: not applicable.

#### Depression

The linear mixed model analysis revealed a significant group effect (*F*=4.906, *P*=.04), a significant time effect (*F*=154.830, *P*<.001), and a significant group × time interaction effect (*F*=26.563, *P*<.001) on the SDS (Table 2). A significant difference was observed between the two groups at T1 (Cohen *d*=–0.474) and T2 (Cohen *d*=–0.541) for the SDS (Figure 2B).

#### Parenting Stress

The results of the linear mixed model analysis showed a significant group effect (*F*=4.083, *P*=.045), a significant time effect (*F*=53.994, *P*<.001), and a significant group × time interaction effect (*F*=38.792, *P*<.001) for parenting distress; a significant group effect (*F*=6.350, *P*=.01), a significant time effect (*F*=34.508, *P*<.001), and a significant group × time interaction effect (*F*=60.743, *P*<.001) for parent-child dysfunctional interaction; and a significant group effect (*F*=8.176, *P*=.005), a significant time effect (*F*=69.315, *P*<.001), and a significant group × time interaction effect (*F*=68.572, *P*<.001) for the PSI-SF total score (Table 2). There was a significant time effect (*F*=11.629, *P*<.001) but no significant group effect (*F*=2.507, *P*=.12) or group × time interaction effect (*F*=.741, *P*=.48) for difficult child (Table 2). A significant difference was observed between the two groups at T1 (Cohen *d*=–0.467) and T2 (Cohen *d*=–1.165) for the PSI-SF total score (Figure 2C).

### Secondary Outcomes

#### Hope

The results showed a significant group effect (*F*=49.276, *P*<.001) and a significant group × time interaction effect (*F*=85.808, *P*<.001) for temporality and future; a significant group effect (*F*=74.080, *P*<.001) and a significant group × time interaction effect (*F*=89.652, *P*<.001) for positive readiness and expectancy; a significant group effect (*F*=50.344, *P*<.001), a significant time effect (*F*=5.690, *P*=.004) and a significant group × time interaction effect (*F*=67.373, *P*<.001) for interconnectedness; and a significant group effect (*F*=105.581, *P*<.001), a significant time effect (*F*=4.787, *P*=.009) and a significant group × time interaction effect (*F*=197.608, *P*<.001) for the HHI total score (Table 2). There were no significant time effects for temporality and future or positive readiness and expectancy (all *P*>.05; Table 2). A significant difference was observed between the two groups at T1 (Cohen *d*=1.968) and T2 (Cohen *d*=2.594) for the HHI total score (Figure 2D).

#### Feasibility

At T2, 90.4% (48.8/54) of all mothers in the intervention group reported that they were extremely satisfied with the WBPT course and 6.7% (3.6/54) indicated that they were slightly satisfied with the WBPT course (Table 3).

At T1, 53.8% (35/65) logged their progress each week and 80.0% (52/65) logged their progress in home training more than 80% of the time during the 12 weeks of the intervention. At T2, 51.8% (29/56) logged their progress each week and 76.8% (43/56) logged more than 80% of the time for 8 weeks after the intervention. In total, 40.0% (26/65) logged their progress each week and 61.5% (40/65) logged their progress more than 80% of the time for all 20 weeks.

**Table 3:** Satisfaction rates of the participants in the intervention group at the T2 assessment (N=54).

Variables	Strongly agree, n (%)	Slightly agree, n (%)	Neutral, n (%)	Slightly disagree, n (%)	Strongly disagree, n (%)
I think that the contents of the WeChat-based parenting training course are very practical.	50 (92.6)	3 (5.6)	1 (1.8)	0 (0.0)	0 (0.0)
I think that the contents of the WeChat-based parenting training course are very comprehensive.	47 (87.0)	5 (9.3)	2 (3.7)	0 (0.0)	0 (0.0)
I think that the WeChat-based parenting training course is very interactive.	48 (88.9)	4 (7.4)	2 (3.7)	0 (0.0)	0 (0.0)
I think that the contents of WeChat-based parenting training course can meet the needs of me and my child.	49 (90.7)	3 (5.6)	2 (3.7)	0 (0.0)	0 (0.0)
Overall, I am satisfied with the WeChat-based parenting training course.	50 (92.6)	3 (5.6)	1 (1.8)	0 (0.0)	0 (0.0)
Average	48.8 (90.4)	3.6 (6.7)	1.6 (2.9)	0 (0.0)	0 (0.0)

## Discussion

### Principal Findings

This was the first study with a pretest-posttest alternative treatment comparison group design to evaluate the impact of the WeChat-based parenting training on mothers with children with autism during the COVID-19 pandemic. The results indicated moderate to large improvements in mothers’ anxiety, depression, parenting stress, and hope levels.

### Effects of the WeChat-Based Parenting Training

In this study, the WeChat-based parenting training that was targeted at mothers with children with autism had statistically significant impacts on anxiety and depression levels, which is consistent with previous studies’ findings [20,33]. Several studies reported that anxiety and depression levels among mothers with children with autism were higher than among mothers with neurotypical children [34-36]. During the COVID-19 pandemic, the public felt anxiety, depression, despair, and many other emotional reactions [37]. Mothers raising children with ASD who might already be under a lot of pressure might suffer from greater psychological distress due to the pandemic. Supporting mothers in the process of caring for children is likely to address the high psychological burden that they face and enhance their overall quality of life [34]. The development of more serious negative maternal outcomes can also be reduced by targeting mothers’ negative thought patterns that are associated with parenting challenges [38]. No-contact consultations by phone, QQ, and WeChat were adopted to help the public cope with the psychological pressure caused by COVID-19 [39]. The WeChat-based parenting training, as one of the no-contact approaches, was conducted to support mothers with children with autism in coping with the pandemic and home training, which may play an important role in alleviating mothers’ anxiety and depression.

The results also revealed that the WeChat-based parenting training was effective in decreasing mothers’ parenting stress. Possible reasons may be as follows. The WeChat-based parenting training, concentrated on the JASPER course, provided an opportunity for mothers to improve their childcare knowledge systems and home training management levels. Furthermore, scenario simulations were provided to help the mothers master specific strategies in response to their children’s communication and behaviors, which is more intuitive and makes parenting knowledge extraction easier. Moreover, online question and answer sessions could also address the mothers’ parenting confusion at the right time. Thus, the dimension scores of parenting distress and parent-child dysfunctional interaction decreased. Another important reason could be that parents who are parenting children with autism face high levels of parenting stress and other negative emotions, both in normal times and in pandemic situations, especially those with recent special service needs [40-42]. The parental psychological intervention course was provided to relieve parenting stress and other negative mental states. ASD has no cure and the WeChat-based parenting training was also unable to change the natures of children with ASD, which is why the dimension scores for difficult child between the two groups had no statistical differences. However, future studies are needed to verify the effect of the WeChat-based parenting training.

The results also demonstrated that the WeChat-based parenting training exerted a significant influence on the hope of the mothers. This may be related to the enhancement of mothers’ childcare knowledge systems and home training management levels. The mothers might have been filled with hope in the process of training children. Hope is defined as the perceived capability to achieve a desired goal and stimulate oneself to follow through using agency thinking [43]. Higher hope is associated with better outcomes in psychological adjustments [43]. The alleviation of psychological pressure and parenting stress during a pandemic may also raise a mother’s hope level.

The possible impact of novelty effects is another interpretation for the moderate to large effect sizes. As there was little access to support for the mental well-being of families at the early stage of the COVID-19 pandemic, the WeChat-based parenting training can be considered a novelty, and even a privilege. Novelty effects may cause participants to be more enthusiastic and pay more attention to interventions, thereby resulting in moderate to large effects [44].

### Feasibility

The mothers’ high-level satisfaction suggested that the WeChat-based parenting training appeared to be a pleasant experience for them and that this approach was acceptable to these mothers with children with autism during the COVID-19 pandemic. In fact, there were no complaints or other problems with the WeChat-based parenting training during the study. The WeChat-based parenting training enabled mothers to conduct progress logging for home training using the WeChat mini-program, which allowed the researcher to observe the participants’ adherence to the intervention and promote researchers to better master the course schedule. The rate of progress logging also indicated an urgent demand for parent training from mothers with children with autism during the outbreak.

### Limitations

There were several limitations in this study. First, due to the pandemic situation, a nonrandomized design was used, which might have led to deviations in the research results. Some scholars point out that findings from nonrandomized trials can be as valid as those of randomized controlled trials, depending on the study quality [45]. In this study, a parallel control group, assessor blinding, and study controls for the baseline characteristics were used to make the study methodology as rigorous as possible. It was rated as high quality using the Newcastle-Ottawa Scale (NOS) [46]. Second, this study was limited to a group of mothers. Further studies should try to explore the differences between fathers and mothers or the differences between only one parent participating in the intervention versus both parents. Third, due to having only one study setting, the generalizability of the findings may be limited. Therefore, future multicenter research with a randomized controlled trial design is necessary. Finally, due to the COVID-19 pandemic, the questionnaire survey was conducted using the online Questionnaire Star platform, which made it impossible to know whether the respondents completed the questionnaire independently and what the respondents’ environments were when answering questions, thereby affecting the judgment of the questionnaire’s quality. Researchers should evaluate the long-term effects of the WeChat-based parenting training using other measurements after the COVID-19 pandemic.

### Conclusions

The WeChat-based parenting training is a promising training method for reducing anxiety, depression, and parenting stress and increasing hope in mothers with children with autism during the COVID-19 pandemic. A rigorous design is needed to further assess the effectiveness of the WeChat-based parenting training in the future.

## Abbreviations

ASD: autism spectrum disorder
DSM-5: Diagnostic and Statistical Manual of Mental Disorders, Fifth Edition
HHI: Herth Hope Index
JASPER: Joint Attention, Symbolic Play, Engagement, and Regulation
PSI-SF: Parenting Stress Index-Short Form
SAS: Self-rating Anxiety Scale
SDS: Self-rating Depression Scale
WBPT: WeChat-based parenting training
